# Evaluation of Cerebral Blood Flow Alterations and Acute Neuronal Damage due to Water-Pipe Smoking

**DOI:** 10.4274/balkanmedj.galenos.2018.2018.0704

**Published:** 2019-02-28

**Authors:** Onur Karakayalı, Uygar Utku, Serkan Yılmaz

**Affiliations:** 1 Department of Emergency Medicine, University of Health Sciences, Derince Training and Research Hospital, Kocaeli, Turkey; 2 Department of Neurology, İstinye University School of Medicine, İstanbul, Turkey

**Keywords:** Acute injury, carboxyhemoglobin, cerebral blood flow, S100 proteins, water pipe smoking

## Abstract

**Background:**

Although water-pipe smoking is a great public health problem, data regarding the acute and chronic effects and the degree of toxin exposure are limited. While water pipe-related malignancy, pulmonary, infectious, cardiac effects, infertility, and biological effects have been described in a meta-analysis, there are no studies in the literature about its neurologic effects.

**Aims:**

To evaluate water pipe-related acute neurological effects and cerebral blood flow through transcranial Doppler ultrasonography and serum S100 calcium binding protein calcium binding protein level measurements.

**Study Design:**

Prospective observational study.

**Methods:**

Vital signs and baseline carboxyhemoglobin and S100 calcium binding protein levels, cerebral flood changes with transcranial Doppler ultrasound were evaluated and recorded before and after water-pipe smoking.

**Results:**

The mean age of the 31 volunteers was 30.61 (±5.67) years, and 24 of them (77.42%) were male. A statistically significant difference was determined in heart rate, oxygen saturation, systolic and diastolic arterial pressure values before and after water-pipe smoking (p<0.001, p=0.035, p=0.009, p=0.021, respectively). Mean carboxyhemoglobin level was 2.68% (±1.68) before, 14.97% (±4.83) after water-pipe smoking (p<0.001). The S100 calcium binding protein level was 25.05 μ/mL (±8.34) at the beginning, 40.71 μ/mL (±14.06) after water-pipe smoking (p<0.001). An increase was determined in peak, and median middle, anterior and posterior cerebral artery blood flow rates, and a decrease was determined in both the pulsatility index and resistivity index values after water-pipe smoking using transcranial Doppler ultrasound.

**Conclusion:**

Cerebral vasodilation develops due to the increase in cerebral blood flow rate and the decrease in pulsatility index, resistivity index values, and the elevation in carboxyhemoglobin, S100 calcium binding protein level indicates that water-pipe smoking leads to neuronal damage in the acute period.

The water pipe (also known as a hookah, shisha) is a traditional tobacco-type pipe, which has been used for more than 400 years. While it is widely used in Middle East countries, its use is gradually increasing among adolescents and young adults worldwide, mainly in the United States, Brazil, and Europe (1). In a study conducted in the United States, 7.2% of young adolescents were found to have used a water pipe during the last month, and 29.5% of these adolescents were determined to have used a water pipe at least once during their lives (2). The main reasons for its use include that it is interesting because of its structure and mechanism, the belief that it is less toxic than cigarettes because of the absence of direct tobacco exposure to the smoke and toxic substances that are eliminated through the water. Water-pipe smoking is increasing today because of this belief. However, studies have indicated that water-pipe smoking exposes subjects to more toxins than cigarette smoking and toxic exposure from the water pipe is equal to 100 cigarettes (3).

Although it is a great public health problem, the degree of toxin exposure and acute/chronic effects are limited. While water pipe-related malignancy, pulmonary, infectious, cardiac effects, infertility, and biological effects have been described in a meta-analysis (4), there are no studies in the literature about its neurologic effects. However, neurological effects such as syncope, dizziness, and headache are common among water-pipe smokers (5,6). These symptoms were reported to result from an elevated carbon monoxide level; however, there is insufficient data in the literature about acute brain damage and changes in cerebral blood flow.

Transcranial Doppler ultrasonography is a non-invasive conventional method that does not lead to radiation exposure and is used for the detection of cerebral blood flow alterations. The potential risk of long-term cerebral effects may be determined by measuring the anterior, middle, and posterior cerebral artery blood flow velocities. Water-pipe smokers are subjected to an ample amount of toxic substances, and the acute effects of these substances on the central nervous system are not known. In particular, elevated carbon monoxide-related neurological symptoms are in the foreground; the carbon monoxide level can be measured with non-invasive tests. However, its measurement alone is not sufficient for determining the direct relationship between neurological effects and chronic effects; the correlation between the carboxyhemoglobin (COHb) level and the severity of intoxication has been reported to be limited and that it cannot be used as a neurological sequel marker (7). Therefore, different biochemical markers are required. The S100 calcium binding protein B (S100b) was reported to be able to be used as an independent marker for the detection of acute injury in different patient groups (8).

The aim of the present study was to evaluate water pipe-related acute neurological effects and cerebral blood flow using transcranial Doppler ultrasonography and serum S100b protein level measurements.

## MATERIALS AND METHODS

### Study design

This prospective observational study was conducted with healthy volunteers at a hookah café. Ethics committee approval was obtained from the local ethics committee. The study was conducted in accordance with the Helsinki Declaration. Written and verbal informed consent was obtained from all participants before the study.

### Volunteer population

The study was conducted with healthy volunteers who had gone to the hookah cafe, and who did not have an acute or chronic disease. No pressure was placed on participants for water-pipe smoking; the potentially harmful effects of water-pipe smoking were explained verbally and in written form. Volunteers who had decided to smoke water pipe despite all warnings were included in the study. Volunteers were excluded from the study if they were under 18 years of age; were pregnant; had coronary artery disease or disorders that could affect the cerebral blood flow such as ischemic stroke, A-V malformation, history of meningitis, or central nervous system disease; had a history of intracranial surgery; currently used anti-arrhythmic, anti-hypertensive, anti-epileptic, or hypnotic-sedative drugs; had a history of alcohol, narcotic or substance use; smoked a water pipe for shorter than 45 min.; had a history of water-pipe smoking during the last 24 hours; or did not agree to participate were excluded from the study.

### Study protocol

Participants who agreed for participation were taken to a closed room in the café before water-pipe smoking and were asked their age, gender, history of smoking (including water-pipe smoking). The basal heart rate, oxygen saturation, and systolic and diastolic blood pressure were measured and recorded. Baseline COHb was measured using a non-invasive Masimo Root Rainbow Set (Masimo Rainbow SET Radical-7 Pulse CO-Oximeter, Masimo Corp., Irvine, CA) device. After basal measurements, volunteers rested for 5 minutes in the sitting position, and transcranial Doppler ultrasonography measurements were made from the temporal window. A 5 mL of venous blood was placed into an EDTA tube for S100b protein measurement and stored at 4 °C in the refrigerator before the second blood samples were drawn.

Volunteers were taken to an open area after baseline measurements and re-evaluated after 45 minutes of water-pipe smoking. They were questioned about the presence of symptoms like headache, dizziness, nausea, vomiting, syncope, palpitation, and dyspnea. Volunteers were taken to the closed room again after 45 minutes of water-pipe smoking to re-measure their vital signs, oxygen saturation, and non-invasive COHb levels with the same device. After vital measurements were taken, transcranial Doppler ultrasonography was performed again. Another 5 mL of venous blood was drawn from peripheral blood into an EDTA tube one hour after the first blood test. All blood samples were transferred to the hospital in blood transfer bags within 30 minutes. Samples were stored at -80 °C after centrifugation. Once the study was completed, all blood samples were examined for S100b in the Elisa device using the S100b Elisa (S100b human plasma) kit (DiaMetra, Spain, 2015, Number: DKO 011, Lot: 4254A).

### Ultrasonography measurements

**Figure 1:**
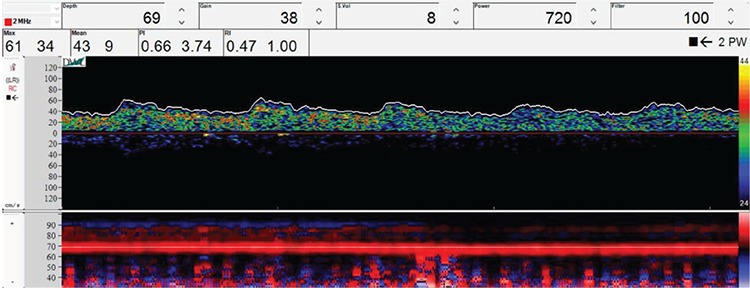
An example of transcranial Doppler ultrasound in the target vessel before water pipe smoking (flow analysis in right middle cerebral artery).

**Figure 2:**
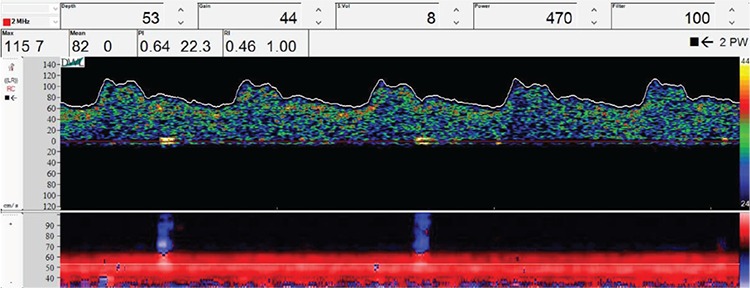
An example of transcranial Doppler ultrasound in the target vessel after water pipe smoking (flow analysis in right middle cerebral artery).

All transcranial Doppler ultrasonography examinations were performed by a single interventional neurologist with 10 years’ experience using the transcranial Doppler ultrasonography device (DWL, MDT-2036, Germany). The transcranial Doppler ultrasonography examination is performed using a 2 MHz frequency ultrasound probe. Measurements were made from the temporal window when volunteers were in the seated position. The peak systolic flow rate, mean flow rate, pulsatility index (PI), and resistivity index (RI) measurements of the right and the left anterior carotid arteries, middle cerebral artery, and posterior cerebral artery were made from the temporal window after the patient had been positioned before water-pipe smoking (Figure 1). Volunteers were transferred to the closed area after 45 minutes of water-pipe smoking, and the same measurements were both taken and recorded again (Figure 2).

Internal carotid artery bifurcation can be found at a depth of 55 to 65 mm and identified by the blood flow away from the probe. The internal carotid artery bifurcation continues as the anterior and the middle cerebral arteries and is an important anatomic marker for locating the anterior circulation. The middle cerebral artery extends laterally and slightly anteriorly from the internal carotid artery root at a depth of 35-55 mm. The middle cerebral artery should be accurate until the trifurcation where the downstream flow becomes bidirectional. The anterior carotid artery, which can be seen at a depth of 60-70 mm, extends medially and anteriorly after the internal carotid artery bifurcation. The anterior carotid artery blood flow is visible at the proximal position. The posterior cerebral artery is displayed in the transtemporal window and usually located 1-2 cm posterior to the internal carotid artery bifurcation in the same plane as the Willis polygon. The posterior cerebral artery can be found posterior and deep to the internal carotid artery  and middle cerebral artery, at a depth of ~60 to 70 mm. Blood flow is toward the probe in the proximal posterior cerebral artery (P1 segment) and away from the probe in the distal posterior cerebral artery (P2 segment) (9).

### Statistical analysis

Statistical analyses were performed using SPSS 15.0 (Chicago, IL). The Shapiro–Wilk test was used to assess the normal distribution of the variables. Non-parametric data were expressed as numbers, percentages, median values, and min-max values, whereas parametric data were expressed as means and standard deviations. Non-parametric categorical parameters were analyzed using the chi-square test, and non-parametric dependent ordinal parameters were analyzed using the Wilcoxon test. Dependent parametric data were analyzed using the paired t-test. Paired t-tests were used to estimate the significance of differences in blood flow velocities, PI, and blood pressure before and after water-pipe smoking. Whenever appropriate, 95% confidence intervals were also calculated, and a p-value less than 0.05 was considered statistically significant.

## RESULTS

During the study period, 31 volunteers were included and evaluated. The mean age of the volunteers was 30.61 (±5.67) years, and 24 of them (77.42%) were male. While 17 volunteers (58.84%) had a history of smoking, 14 volunteers (45.16%) had a history of regular water-pipe smoking. Once the symptoms after water-pipe smoking were analyzed, 18 volunteers (58.1%) were observed to have no symptoms, 9 volunteers (29%) had headache, 3 of them (9.7%) had dizziness, and 1 of them (3.2%) had dyspnea.

The heart rate, oxygen saturation, systolic and diastolic arterial pressure values before and after water-pipe smoking are summarized in Table 1. A statistically significant difference was found in all parameters (p<0.001, p=0.035, p=0.009, p=0.021, respectively). The median COHb level was 3% (min: 0-max: 6) before and 14% (min: 9-max: 26) after water pipe smoking.Water pipe smoking-related changes of COHb were statistically significant (p<0.001) (Table 1). The median S100b protein level was 22.3 μ/mL (min: 7.28-max: 48.72) at the beginning and 36,8 μ/mL (min: 15.19-max: 68.67) after water pipe smoking. A statistically significant difference was found in S100b protein after water-pipe smoking (p<0.001) (Table 1). When the relationship between S100b protein values of subjects who smoked water pipe regularly and for the first time was analyzed, it was determined as 24.76 μ/mL (±8.19) in subjects who regularly smoked water pipe and 25.28 μ/mL (±8.7) in subjects who smoked it for the first time. No statistically significant difference was determined between subjects who regularly smoked water pipe and subjects who smoked it for the first time regarding baseline S100b protein levels (p=0.875).When S100b protein level alterations were analyzed before, and after water-pipe smoking, the increase in S100b protein levels was 19.57 μ/mL (±13.5) in subjects who smoked water pipe chronically and 12.69 μ/mL (±6.48) in subjects who smoked it for the first time. No statistically significant difference was found between the two groups (p=0.085). While the mean baseline COHb level was 3.12% (±1.83), S100b protein level was 27.04 μ/mL (±6.71) in smoking volunteers; these values were 2.14 (±1.35) and 22.64 μ/mL ±9.68) in non-smokers. No statistically significant difference was found between smokers and non-smokers regarding both baseline COHb and S100b levels (p=0.415, p=0.463, respectively).

The peak systolic flow rate in the anterior carotid artery, middle cerebral artery and posterior cerebral artery, and the mean flow rate, PI, and RI values of volunteers are summarized in Table 2. An increase in blood flow rate was determined in the left, and the right in peak and mean middle cerebral artery, anterior cerebral artery, and posterior cerebral artery, whereas a decrease was determined in both the PI and RI values after water-pipe smoking. A statistically significant difference was determined in all measurements, except for the average left middle cerebral artery (p=0.072), left anterior cerebral artery PI (p=0.137), and left anterior cerebral artery RI (p=0.085) values. The mean transcranial Doppler ultrasonography measurement values before and after water-pipe smoking and the p values are presented in Table 2. No statistically significant difference was found between chronic cigarette or water-pipe smokers and subjects who smoked for the first time regarding baseline peak systolic flow rate, mean blood flow rates, and both PI and RI values (p=0.242, p=0.105, respectively).

## DISCUSSION

Our study revealed an increase in carbon monoxide level, heart rate, systolic and diastolic blood pressure, and a decrease in oxygen saturation due to water-pipe smoking. While water-pipe smoking was found to lead to an increase in peak and mean anterior carotid artery, middle cerebral artery, and posterior cerebral artery blood flow rates, it was observed to lead to a decrease in both PI and RI values. A statistically significant increase was found in S100b protein levels, which is used for the detection of water pipe-related cerebral injury.

Water-pipe smoking was shown to lead to an increase in systolic blood pressure, diastolic blood pressure, and heart rate (10,11). A mean of 45 min of water-pipe smoking was shown to lead to a 6 bpm increase in heart rate (12), 16 bpm increase in heart rate, 6.7 mmHg increase in systolic blood pressure, and 4.4 mmHg increase in diastolic blood pressure (13). The results of our study are similar to those in the literature; oxygen saturation was also observed to decrease in volunteers. We consider that the reduction in oxygen saturation results from hypoxia. These hemodynamic changes are believed to result from the elevated nicotine level leading to an increase in norepinephrine, epinephrine, and vasopressin or through its direct effect on the endothelium and sympathetic nervous system activation.

Previous studies have shown a positive correlation between water-pipe smoking and COHb level. In the study conducted by Zahran et al. (14) with 1832 volunteers who chronically smoked cigarettes and water pipe, the COHb level was shown as 6.47±2.7 among cigarette smokers and 10.06±2.5 in water pipe smokers. Al-Moamary et al. (15) reported a 30% increase in the COHb level after water-pipe smoking compared with healthy volunteers; Levant et al. (16) found this value to be 20.8% and Yıldırım et al. (17) determined this level as 23.7% (minimum-maximum: 6%-44%). The long-term inhalation of the smoke of the coal used for firing the tobacco in hookahs prepared by conventional methods leads to an increase in COHb levels. Patients presenting to the emergency room because of COHb elevation-related symptoms such as headache, nausea, vomiting, dizziness, seizures, and syncope have been reported in the literature (18,19). Similarly, a statistically significant elevation in COHb and related neurological symptoms were detected among subjects who smoked water pipe in our study.

Transcranial Doppler ultrasonography is the most common non-invasive method for assessment of cerebral blood flow. Smoking-related cerebral blood flow alterations have been reported in the literature despite the absence of studies about water-pipe smoking. Varying degrees of distal blood flow increases were shown in intracranial vessels (anterior carotid artery, middle cerebral artery, posterior cerebral artery), and a decrease was shown in both PI and RI as a result of smoking a single cigarette (20). In the posterior cerebral artery and middle cerebral artery, the end-diastolic volume increased (7.8% in the posterior cerebral artery, 14.4% in the middle cerebral artery) and the peak systolic volume increased in the anterior carotid artery (1.1%) and the middle cerebral artery (7.5%). The average rising end-diastolic volume (14%) in the middle cerebral artery is higher than the peak systolic volume (7.5%). The PI of the middle cerebral artery decreased at the same time. These results show that smoking causes a reduction in vascular resistance and a rise in cerebral blood flow. Cardiac output directly affected peak systolic volume but end-diastolic volume is thought to increase with a decrease in peripheral vascular resistance (21). The increase in peak systolic volume in our participants after smoking showed that cigarette smoking increased cardiac output and that combined with low impedance indices should support cerebral blood flow. This result is supported by the increase in both blood pressure and heart rate in our participants after smoking. The increase in blood pressure and the decrease in PI and RI values were reported to to play a potential role in chronic diseases like hypertensive encephalopathy and Alzheimer’s disease in the long term due to vascular resistance (22). In our study, an increase was determined in the peak systolic and end-diastolic blood flow rate, and a decrease was determined in both the PI and RI values. In general, our results show that the immediate effects of cigarette smoking are reduced cardiac output by reducing peripheral cerebrovascular impedance and possibly promoting vasospasm of the middle cerebral artery and/or other basal cerebral arteries.

S100b protein is present in high concentrations in astroglial cells and white matter besides being produced in Schwann cells and peripheral nerve cells. S100b protein rapidly transfers to the cerebrospinal fluid and secondarily to the circulation if these cells are damaged, and the blood-brain barrier is compromised. S100b protein is reported to be effective for predicting neurological brain damage, which may develop in hypoxic brain damage (23). S100b protein is recommended for the rapid detection of neuronal damage resulting from traumatic brain injury, stroke, or subarachnoid hemorrhage, and synthetic cannabinoid use (24,25,26,27). Carbon monoxide elevation-related brain damage is assumed to develop secondary to hypoxia. However, inflammation and oxidative stress may also lead to neuronal damage (7). In our study, the S100b protein level was observed to increase significantly due to water-pipe smoking. The absence of a significant difference between chronic cigarette or water-pipe smokers and subjects who smoked for the first time regarding COHb and S100b protein levels has indicated that the effect is both acute and not associated with chronic use. Although no studies are available in the literature investigating water pipe smoking-related neurological damage, neuronal damage develops due to indirect carbon monoxide elevation. Carcinogenic aromatic hydrocarbons, nitric oxide, and nicotine may also lead to neuronal damage through oxidative stress. Despite the limited number of studies about oxidative stress, we suspect that toxic substances in hookahs may act through a mechanism similar to that in cigarettes, based on the study of Golbidi et al. (28) who investigated the effects of smoking on oxidative stress.

There was some limitations of this study. Our study was a single-center study conducted with a small number of volunteers; furthermore, the small number of female subjects and narrow age range led to a limitation for making a general conclusion. The study group was not distributed homogeneously because of the history of cigarette or water-pipe smoking in a subset of the volunteers. However, we consider that this limitation is not significant as there was no significant differences between the smoking and the non-smoking groups, and subjects who had a history of water-pipe smoking and who smoked water pipe for the first time regarding baseline COHb and S100b protein levels. Although all volunteer groups constantly smoked water pipe for 45 minutes, the duration, number, and depth of inhalations were not constant. However, an increase was determined in the COHb and S100b protein levels, an increase in cerebral blood flow, and a decrease in both PI and RI values compared with baseline values.

While water-pipe smoking is an important public health problem, it is believed to be less toxic than cigarettes. Many neurological symptoms may develop because of water-pipe smoking, and we think that these symptoms develop due to the elevation in the COHb level. Cerebral vasodilation develops due to the increase in cerebral blood flow rate and the decrease in PI and RI values. In addition, the elevation in S100b level indicates that water-pipe smoking leads to neuronal damage in the acute period. We think that progressive and chronic effects may develop in volunteers in the long term because of both progressive neuronal damage and decreased vascular resistance. Therefore, we believe that all government and community service organizations should direct the same efforts against water-pipe smoking as they did against cigarette smoking in the past.

## Figures and Tables

**Table 1:**
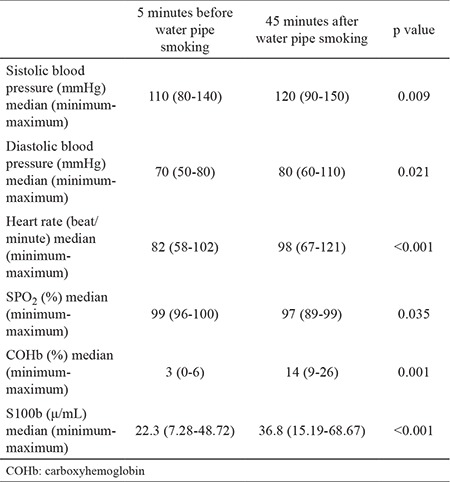
Characteristics of vital signs, COHb levels and S100b levels on before and after water pipe smoking

**Table 2:**
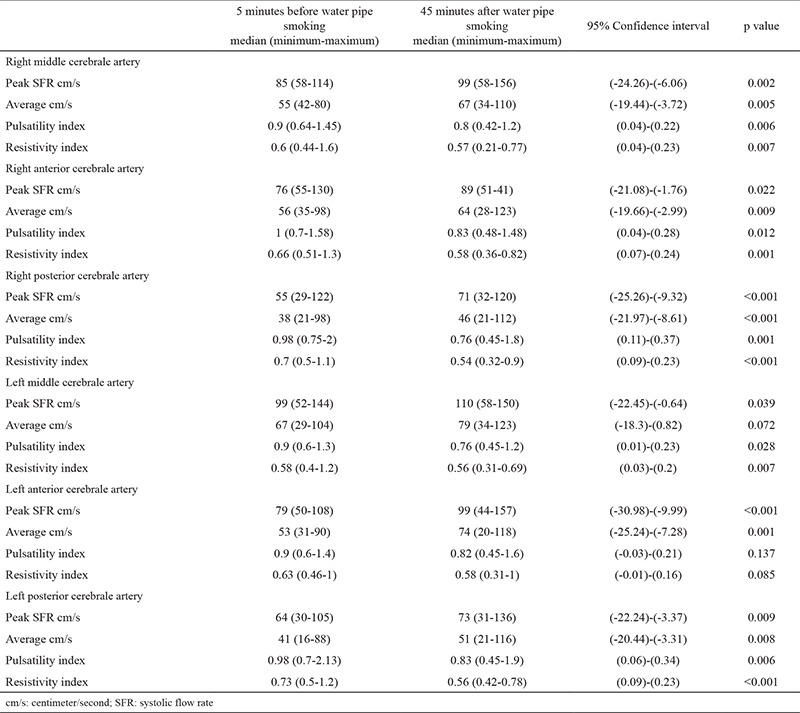
Transcranial Doppler measurement before and after water pipe smoking
